# Athletic identity and sport commitment in athletes after anterior cruciate ligament reconstruction who have returned to sports at their pre-injury level of competition

**DOI:** 10.1186/s13102-021-00264-6

**Published:** 2021-04-07

**Authors:** Shunsuke Ohji, Junya Aizawa, Kenji Hirohata, Sho Mitomo, Takehiro Ohmi, Tetsuya Jinno, Hideyuki Koga, Kazuyoshi Yagishita

**Affiliations:** 1 Clinical Center for Sports Medicine and Sports Dentistry, Tokyo Medical and Dental University, 1-5-45 Yushima, Bunkyo-ku, Tokyo, 113-8519 Japan; 2 Department of Physical Therapy, Juntendo University, 3-2-12 Hongo, Bunkyo-ku, Tokyo, 113-0033 Japan; 3 Department of Orthopaedic Surgery, Dokkyo Medical University Saitama Medical Center, 2-1-50 Minami-Koshigaya, Koshigaya, Saitama, 343-8555 Japan; 4 Department of Joint Surgery and Sports Medicine, Tokyo Medical and Dental University, 1-5-45 Yushima, Bunkyo-ku, Tokyo, 113-8519 Japan

**Keywords:** Athletic identity, Anterior cruciate ligament reconstruction, Sport commitment, Return to sport, return to performance

## Abstract

**Background:**

This study aimed to determine the relationships between athletic identity and sport commitment and return to sports (RTS) status in athletes after anterior cruciate ligament reconstruction (ACLR).

**Methods:**

Thirty-nine participants post-ACLR (8–24 months) were included in this cross-sectional study. Measures included the athletic identity measurement scale and sport commitment scale. In addition, we measured kinesiophobia and psychological readiness using the Tampa Scale for Kinesiophobia and ACL-Return to sport after injury scale. The subjects were categorized into Yes-RTS or No-RTS based on two questions to determine whether they were returning to sport at the same level of competition as before the injury. A Chi-squared test, Fisher’s exact test, unpaired t-test, and Mann-Whitney’s U test were used to analyze the data.

**Results:**

The Yes-RTS group had significantly higher scores on the athletic identity measurement scale (*P* = 0.023, effect size [ES] = − 0.36), sport commitment scale (*P* = 0.027, ES = − 0.35), and ACL-Return to sport after injury scale (*P* = 0.002, ES = − 0.50) and significantly lower Tampa Scale for Kinesiophobia scores (*P* = 0.014, ES = − 0.39) compared to the No-RTS group.

**Conclusion:**

Athletes who returned to sports at the same level of competition as before the injury had higher athletic identity and sport commitment and lower kinesiophobia compared to those who did not return to sports at the same level of competition. These self-beliefs regarding sport may play an important role in post-ACLR athletes’ RTS.

## Background

Most athletes with an anterior cruciate ligament (ACL) injury undergo ACL reconstruction (ACLR) with the goal to return to sport (RTS) at the same level of competition as before the injury [1] but only 63% of athletes are able to achieve this [2]. Multiple factors are associated with RTS after ACLR, including injury site and surgical technique, physical functioning, and psychology [3]. The influence of psychological factors is particularly large in athletes in the RTS phase [2, 4]. Compared to before the injury, athletes RTS after ACLR have the following psychological characteristics: weak kinesiophobia (fear of re-injury and movement) [5–7], high self-efficacy [6], high self-esteem [8], and high psychological readiness to RTS [6, 9, 10].

Recently, athletic identity and sport commitment have been recognized as important psychological variables that could be related to RTS status post-ACLR [4, 8, 11, 12]. Athletic identity is the sport-specific component of an individual’s self-concept and is the extent to which an individual identifies with the athletic role [13]. Post-ACLR athletes with a higher degree of athletic identity show greater adherence to rehabilitation [14]. Sport commitment is defined as a psychological state representing the desire and resolve to continue participating in a particular athletic program, specific sport, or sports in general [15]. Athletes who have suffered severe injuries, including ACL injuries, can continue being committed to RTS through sport commitment [11].

Based on these studies, it is expected that athletic identity and sport commitment would be associated with RTS status in post-ACLR athletes. However, no previous study has quantified the relationship between athletic identity and sport commitment and RTS status following ACLR. Therefore, the purpose of this study was to determine the relationships between athletic identity and sport commitment, and RTS status in athletes after ACLR. We hypothesized that post-ACLR athletes who have returned to sports at their pre-injury competition level have higher athletic identity and sport commitment scores compared to athletes who have not returned to sports.

## Methods

### Participants

Participants who had undergone primary ACLR between August 2015 and May 2019 were included if they met the following inclusion criteria: (1) they were 16 to 45 years old at the time of measurement [10, 16]; (2) their sport participation estimated with a modified Tegner activity scale [17] was ≥5 before ACL injury; (3) it had been 8–24 months since the surgery [18]; and (4) they had indicated an intention to RTS before surgery. Participants were excluded if they had an ACL injury to the contralateral knee or ACL reinjury to the reconstructed knee; had a complication that affects RTS; had not participated in sports for social reasons such as pregnancy or employment; had a cartilage injury requiring surgery; and had difficulty in follow-up until RTS. The sample size was analyzed using G*Power software [19]. The minimum sample size was calculated to be 38 patients in total, referring to the effect size determined from previous studies [6, 9, 10, 20, 21] analyzing group differences in ACL-Return to sport after injury scale (ACL-RSI) and Tampa Scale for Kinesiophobia (TSK) scores (effect size = 0.96, alpha = 0.05, power = 0.80, two-tailed). All surgeries were performed by orthopedic surgeons specialized in knee joint. The autograft sources were hamstrings or bone-patellar tendon-bone. The surgery technique and postoperative rehabilitation protocol were based on previous research [22]. Jogging started 3 months post-ACLR, and the running speed was gradually increased. Sports participation was allowed by a doctor when the following were achieved: it was at least 6 months after surgery; the limb symmetry index (LSI) on the single-leg hop for distance was > 90%; and the LSI of isokinetic knee flexion and extension strength was > 85%, as measured with an Isokinetic Dynamometer (BIODEX System 4, BIODEX Medical Inc., Shirley, NY) at 60°/s and 180°/s).

### Procedures

This was a cross-sectional study completed in a single center. Demographic, injury, and surgical information were collected from medical records. Sport type, participation level, and psychological variables were collected using a questionnaire. Sport type was categorized as collision, contact, limited contact, and noncontact based on a previous study [23]. Participation level was categorized as recreation, competitive, and elite based on a previous study [8]. Ethical approval was obtained from the Ethics Committee (approval number: M2016–197). All subjects provided written informed consent before participation. The Strengthening the Reporting of Observational Studies in Epidemiology (STROBE) Statement was used as guidance when reporting the design of this study [24].

#### Psychological variables

This study measured athletic identity and sport commitment as psychological variables. In addition, we measured kinesiophobia and psychological readiness using standard psychological measures that have already been found to be associated with RTS status after ACLR [5, 6, 10].

Athletic identity was assessed with the Athletic Identity Measurement Scale (AIMS) [13]. The AIMS is a 10-item questionnaire where responses are on a seven-point Likert scale that ranges from 1 (strongly disagree) to 7 (strongly agree). Total scores range from 7 to 49, with higher scores indicating stronger athletic identity. The Japanese version of the AIMS was used, which has good internal consistency and good criterion-related and construct validity [25].

Sport commitment was measured using the Sport Commitment Scale (SCS) [15]. The Japanese version of the SCS [26] was used. This scale is a self-report inventory measuring an athlete’s psychological desire to continue sport participation. SCS is a six-item questionnaire where responses are provided on a five-point Likert scale. Total scores range from 6 to 30, with higher scores indicating greater sport commitment. The SCS has good reliability (internal consistency, reproducibility) and validity (construct and criterion-related validity) [26].

Kinesiophobia was measured using the TSK [27]. The Japanese version of the TSK was used [28]. The TSK is a 17-item questionnaire with a four-point Likert scale. Total scores range from 17 to 68, with higher scores indicating greater kinesiophobia. The TSK has good internal consistency [27].

The ACL-RSI is designed to measure comprehensive psychological readiness to RTS after ACL injury or reconstruction surgery [29]. It is a 12-item questionnaire and includes three domains: emotions, confidence in performance, and risk appraisal. Scores for each domain are summed and averaged for a total score between 0 and 100. Higher scores indicate greater psychological readiness to RTS. The Japanese version of the ACL-RSI was used; it has good internal consistency, construct validity, and reliability [30].

#### RTS status

To determine RTS status, all subjects responded to two questions, one of which was a continuous variable and the other was dichotomous. The continuous variable question was assessed using post-operative subjective athletic performance (POSAP) on a scale of 0–100% [22]. In the dichotomous question, the participants were asked to answer “Yes” or “No” if they were returning to the same level of sports as before the injury [5, 21, 31–33]. The subjects who answered > 80% for the PoSAP and “Yes” to the dichotomous question were included in the Yes-RTS (YRTS) group. The No-RTS (NRTS) group included those who met none or only one of the criteria.

### Statistical analysis

The normality of the distribution of each variable was determined by a histogram and the Shapiro–Wilk normality test. The differences between the NRTS and YRTS groups in demographic data and psychological variables were analyzed using the chi-squared test, Fisher’s exact test, unpaired t-test, and Mann-Whitney’s U test. The effect sizes (chi-squared test or Fisher’s exact test = φ coefficient, Cramer’s V, t-test = Cohen’s d, Mann-Whitney’s U test = r) were also calculated for each variable. Psychological variables may be influenced by activity levels and the months since the surgery. Thus, Spearman’s rank correlation coefficient (ρ) was calculated between the modified Tegner activity scale and the months since the surgery, and the psychological variables. The a priori α level was 0.05. Data were analyzed using SPSS Ver. 21.0 (IBM Corp, Armonk, NY).

## Results

**Fig. 1:**
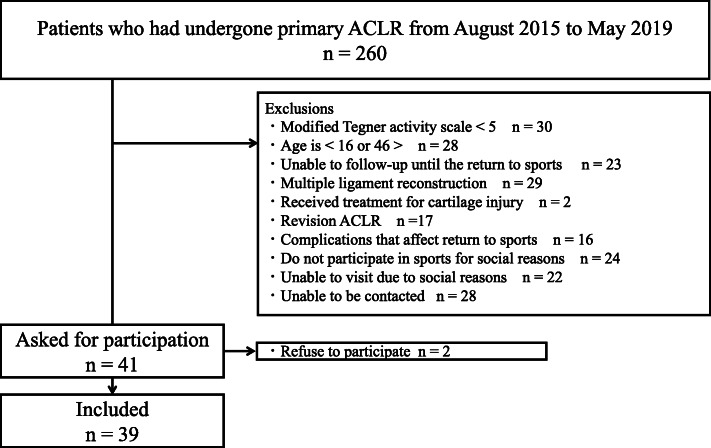
Participant flow chart. ACLR, anterior cruciate ligament reconstruction

Forty-one participants met the criteria for this study, but two refused to participate (Fig. 1). Therefore, 39 participants were included in the analysis. The demographic information of these athletes is presented in Table 1. In total, 16 athletes (41%) were assigned to the NRTS group, and 23 athletes (59%) were assigned to the YRTS group. Four subjects responded “Yes” to the dichotomous question but had a PoSAP ≤80%. The lowest PoSAP for the YRTS group was 85%.

**Table 1:** Demographic variable distributions

	NRTS (*n* = 16)	YRTS (*N* = 23)	*P* value	Effect size
Age, y^a^	23.0 (20.3)	20.0 (4.0)	0.044	− 0.32
Sex (female/male), n	5/11	12/11	0.167	0.21
Body mass index^a^	25.9 (9.0)	22.3 (2.8)	0.116	−0.25
Injury type (contact/non-contact), n	4/12	5/18	0.554	0.04
Months from surgery^a^	11.5 (5.5)	12.0 (5.0)	0.635	−0.08
Days from injury to surgery^a^	68.5 (41.0)	81.0 (100.0)	0.607	−0.08
Graft type (hamstring/BTB), n	14/2	20/3	0.674	0.01
Meniscus repair (yes/no), n	12/4	16/7	0.500	0.06
Pre-injury modified Tegner scale^a^	8.0 (2.5)	8.0 (1.0)	0.551	−0.10
Sports type (Collision/Contact/Limited contact/Noncontact), n	4/6/1/5	9/8/1/5	0.805	0.16
Participation level (Recreation/Competitive/Elite), n	4/11/1	1/17/5	0.098	0.35

^a^Median (inter interquartile range)

*NRTS* no-return to sports, *YRTS* yes-return to sports, *BTB* bone-patellar tendon-bone

There were no significant differences in demographic information, surgical information, or modified Tegner activity scale scores, sports type, and participation level other than age (*P* = 0.044). The data for the psychological variables are shown in Table 2. The YRTS group had a higher AIMS score (*P* = 0.023), SCS score (*P* = 0.027), and ACL-RSI score (*P* = 0.002) than the NRTS group. The YRTS group had a lower TSK score (*P* = 0.014) than the NRTS group.

**Table 2:** Group differences in psychological variables

Psychological variables score	NRTS (*n =* 16)	YRTS (*N =* 23)	*P* value	Effect size
AIMS	35.0 (10.5)	40.0 (8.0)	0.023	−0.36
SCS	20.5 (9.5)	26.0 (5.0)	0.027	−0.35
TSK	36.5 (6.3)	32.0 (11.0)	0.014	−0.39
ACL-RSI	60.8 (34.8)	85.0 (16.7)	0.002	−0.50

(median (interquartile range))

*NRTS* no-return to sports, *YRTS* yes-return to sports, *AIMS* athletic identity measurement scale, *SCS* sport commitment scale, *TSK* Tampa scale for kinesiophobia, *ACL-RSI* anterior cruciate ligament-return to sport after injury scale

The correlations between the modified Tegner activity scale and months since the surgery and the psychological variables are shown in Tables 3 and 4. No significant correlations were found for each variable.

**Table 3:** Correlation between activity level, months from surgery and psychological variables in “NO” return to sports

*n* = 16	mTegner		Months from surgery	
	ρ	*P* value	ρ	*P* value
AIMS	0.40	0.125	−0.23	0.385
SCS	−0.13	0.646	−0.09	0.728
TSK	0.18	0.497	0.01	0.965
ACL-RSI	−0.30	0.267	0.29	0.273

mTegner presents the modified Tegner activity scale

*AIMS* athletic identity measurement scale, *SCS* sport commitment scale, *TSK* Tampa scale for kinesiophobia, *ACL-RSI* anterior cruciate ligament-return to sport after injury scale

**Table 4:** Correlation between activity level, months from surgery and psychological variables in “YES” return to sports

*n* = 23	mTegner		Months from surgery	
	ρ	*P* value	ρ	*P* value
AIMS	< 0.01	0.985	0.19	0.399
SCS	0.09	0.685	0.08	0.735
TSK	−0.37	0.083	0.28	0.203
ACL-RSI	−0.13	0.558	−0.27	0.221

mTegner presents the modified Tegner activity scale

*AIMS* athletic identity measurement scale, *SCS* sport commitment scale, *TSK* Tampa scale for kinesiophobia, *ACL-RSI* anterior cruciate ligament-return to sport after injury scale

## Discussion

The results of this study showed that the YRTS group had significantly higher AIMS, SCS, and ACL-RSI scores and significantly lower TSK scores compared to the NRTS group. Consequently, the results of the present study supported our hypothesis.

A meta-analysis examining the RTS rates of post-ACLR athletes reported that approximately 63% of athletes were able to RTS at the same level of competition as before the injury [2]. The YRTS group in this study included approximately 59% of the participants so the RTS rate for the study population did not differ from previous meta-analyses.

The dichotomous question evaluating RTS as Yes/No alone may overestimate the RTS [22]. Therefore, in the present study, a matrix of the dichotomous question and the PoSAP was used as a measure of RTS. Thereby, four of the 27 participants (15%) who answered ‘Yes’ to the dichotomous question had PoSAP scores under 80%, and they were included in the NRTS. In this study, those who returned to sport closer to their pre-injury status were selected as YRTS.

The demographic data showed that those included in the NRTS group were significantly older than those included in the YRTS group. This supports the findings of previous studies [5, 10]. The wide range of age distribution in the NRTS group in this study may have affected the results. Notwithstanding, several studies have reported that age is not related to RTS status [20, 34] but no consensus has been reached regarding this. Future studies may be necessary to gather more detailed evidence.

The results of the present study showed that those with YRTS had significantly higher AIMS scores than those with NRTS. Athletic identity has been identified as one of the psychological factors associated with RTS after ACLR [4, 8]. However, no previous studies have examined these associations quantitatively. In a cohort study, Brewer et al. [14] reported a positive correlation between preoperative AIMS scores and adherence to rehabilitation (home exercise and self-care) after ACLR. Brewer et al. [35] showed in another cohort study that AIMS scores were reduced in those who did not progress sufficiently in rehabilitation between 6 and 12 months after ACLR. These studies suggest that athletic identity in post-ACLR athletes may influence rehabilitation progression toward RTS. These characteristics may be reflected in the results of this study.

The findings of this study showed that those with YRTS had significantly higher SCS scores than those with NRTS. Until now, no previous studies had quantified the association between RTS status and SCS after ACLR. In semi-structured interviews with post-ACLR athletes, Mahood et al. [12] demonstrated the importance of commitment as one of the driving reasons to RTS. Inigo et al. [11] provided interesting information in response to the question regarding why injured athletes, including post-ACLR athletes, continue to gravitate towards RTS despite the increased potential for future relapses and complications. This interview study showed that a commitment to sport (enjoyment of sport, valuable opportunities, personal investment, social constraints, and social support) enables severely injured athletes to continue to commit to RTS. Thus, it is considered that athletes after ACLR can engage in rehabilitation toward RTS through sports commitment.

The AIMS and SCS show different characteristics depending on the activity level and postoperative period [26, 35, 36]. To consider the confounding effects of these variables on outcomes, the present study analyzed the correlations between each psychological measure and the modified Tegner activity scale and months from surgery but there were no significant correlations. The results of this study show an association between RTS status and athletic identity and sport commitment, regardless of the activity level and postoperative period.

The results of this study showed that the YRTS group had lower TSK scores and higher ACL-RSI scores than the NRTS group. Excessive kinesiophobia and lack of psychological readiness for RTS are the major psychological factors affecting RTS status in post-ACLR athletes [6, 9, 10, 21]. The results of the present study support these findings and provide evidence that kinesiophobia and psychological readiness are associated with RTS status.

### Clinical implications

The minimum time from post-ACLR to RTS is 6 months and, in recent years, it has been recommended to extend the duration of RTS to reduce the risk of re-injury [18, 37]. During such a long rehabilitation period, patients may experience a loss of sport commitment and athletic identity. Additionally, those with significant declines in these variables may need to consider collaborating with a psychologist [8]. In the rehabilitation of post-injury athletes, it is important to set suitable goals and to explain the reasons for exercising to maintain patient and athlete adherence and commitment to rehabilitation [38, 39]. Rehabilitation milestones after ACLR are jogging, running, partial participation in competition, and RTS [40, 41]. We should try to prevent the loss of the patient’s sport commitment and athletic identity by always explaining to them the rationale and purpose of these milestones and what treatment is needed to achieve them.

### Limitations of this study

There are several limitations to this study. First, it was conducted in a single center with a small sample size, so results should be generalized with caution. Second, this study only showed a cross-sectional association between RTS status and psychological scales; the causal relationship between the results is unknown. Although no statistical association was found between the psychological variables and months since surgery in this study, we included athletes whose psychological scores could vary (8–24 months) in the analysis. Third, the present study analyzed subjects who met the criteria for sport participation but did not analyze physical factors that may affect RTS. Future research is necessary to clarify the relationship between athletic identity and sport commitment and RTS status during rehabilitation in a cohort study and to clarify the degree of influence of each factor using a multivariate analysis, including physical functioning.

## Conclusion

Athletes who were able to RTS at their pre-injury level of competition showed higher scores on the athletic identity and sport commitment questionnaires than those who did not. These self-beliefs regarding sport participation may play an important role in post-ACLR athletes’ RTS.

## Data Availability

The datasets used and analyzed during the current study are available from the corresponding author on reasonable request. Data that supports the findings (demographic information and graft types) were collected from medical records, and so are not publicly available. Data are however available from the authors upon reasonable request and with permission of Tokyo Medical and Dental University.

## Abbreviations

AIMS: Athletic Identity Measurement Scale
ACL: Anterior cruciate ligament
ACLR: Anterior cruciate ligament reconstruction
ACL-RSI: ACL-Return to sport after injury scale
NRTS: No-RTS
PoSAP: Postoperative subjective athletic performance
RTS: Return to sports
SCS: Sport Commitment Scale
TSK: Tampa Scale for Kinesiophobia
YRTS: Yes-RTS

## References

[1] Bjordal JM, Arnły F, Hannestad B, Strand T (1997). Epidemiology of anterior cruciate ligament injuries in soccer. Am J Sports Med.

[2] Ardern CL, Webster KE, Taylor NF, Feller JA (2011). Return to sport following anterior cruciate ligament reconstruction surgery: a systematic review and meta-analysis of the state of play. Br J Sports Med.

[3] Czuppon S, Racette BA, Klein SE, Harris-Hayes M (2014). Variables associated with return to sport following anterior cruciate ligament reconstruction: a systematic review. Br J Sports Med.

[4] Everhart JS, Best TM, Flanigan DC (2015). Psychological predictors of anterior cruciate ligament reconstruction outcomes: a systematic review. Knee Surg Sports Traumatol Arthrosc.

[5] Lentz TA, Zeppieri G, George SZ, Tillman SM, Moser MW, Farmer KW, Chmielewski TL (2015). Comparison of physical impairment, functional, and psychosocial measures based on fear of reinjury/lack of confidence and return-to-sport status after ACL reconstruction. Am J Sports Med.

[6] Ardern CL, Österberg A, Tagesson S, Gauffin H, Webster KE, Kvist J (2014). The impact of psychological readiness to return to sport and recreational activities after anterior cruciate ligament reconstruction. Br J Sports Med.

[7] Ardern CL, Taylor NF, Feller JA, Whitehead TS, Webster KE (2013). Psychological responses matter in returning to preinjury level of sport after anterior cruciate ligament reconstruction surgery. Am J Sports Med.

[8] Christino MA, Fantry AJ, Vopat BG (2015). Psychological aspects of recovery following anterior cruciate ligament reconstruction. J Am Acad Orthop Surg.

[9] Langford JL, Webster KE, Feller JA (2009). A prospective longitudinal study to assess psychological changes following anterior cruciate ligament reconstruction surgery. Br J Sports Med.

[10] King E, Richter C, Jackson M, Franklyn-Miller A, Falvey E, Myer GD, Strike S, Withers D, Moran R (2020). Factors influencing return to play and second anterior cruciate ligament injury rates in level 1 athletes after primary anterior cruciate ligament reconstruction: 2-year follow-up on 1432 reconstructions at a single center. Am J Sports Med.

[11] Iñigo MM, Podlog L, Hall MS (2015). Why do athletes remain committed to sport after severe injury? An examination of the sport commitment model. The Sport Psychologist.

[12] Mahood C, Perry M, Gallagher P, Sole G (2020). Chaos and confusion with confidence: managing fear of re-injury after anterior cruciate ligament reconstruction. Phys Ther Sport.

[13] Brewer BW, Van Raalte JL, Linder DE (1993). Athletic identity: Hercules' muscles or Achilles heel?. Int J Sport Psychol.

[14] Brewer BW, Cornelius AE, Van Raalte JL, Petitpas AJ, Sklar JH, Pohlman MH, Krushell RJ, Ditmar TD (2003). Age-related differences in predictors of adherence to rehabilitation after anterior cruciate ligament reconstruction. J Athl Train.

[15] Scanlan TK, Carpenter PJ, Simons JP, Schmidt GW, Keeler B (1993). An introduction to the sport commitment model. J Sport Exerc Psychol.

[16] Patel NK, Sabharwal S, Hadley C, Blanchard E, Church S (2019). Factors affecting return to sport following hamstrings anterior cruciate ligament reconstruction in non-elite athletes. Eur J Orthop Surg Traumatol.

[17] Fältström A, Hägglund M, Kvist J (2013). Patient-reported knee function, quality of life, and activity level after bilateral anterior cruciate ligament injuries. Am J Sports Med.

[18] Nagelli CV, Hewett TE (2017). Should return to sport be delayed until 2 years after anterior cruciate ligament reconstruction? Biological and Functional Considerations. Sports Med.

[19] Faul F, Erdfelder E, Lang AG, Buchner A (2007). G*power 3: a flexible statistical power analysis program for the social, behavioral, and biomedical sciences. Behav Res Methods.

[20] Webster KE, McPherson AL, Hewett TE, Feller JA (2019). Factors associated with a return to Preinjury level of sport performance after anterior cruciate ligament reconstruction surgery. Am J Sports Med.

[21] Lentz TA, Zeppieri G, Tillman SM, Indelicato PA, Moser MW, George SZ, Chmielewski TL (2012). Return to preinjury sports participation following anterior cruciate ligament reconstruction: contributions of demographic, knee impairment, and self-report measures. J Orthop Sports Phys Ther.

[22] Ohji S, Aizawa J, Hirohata K, Ohmi T, Koga H, Okawa A, Jinno T, Yagishita K (2020). The gap between subjective return to sports and subjective athletic performance intensity after anterior cruciate ligament reconstruction. Orthop J Sports Med.

[23] Montalvo AM, Schneider DK, Webster KE, Yut L, Galloway MT, Heidt RS, Kaeding CC, Kremcheck TE, Magnussen RA, Parikh SN, Stanfield DT, Wall EJ, Myer GD (2019). Anterior cruciate ligament injury risk in sport: a systematic review and meta-analysis of injury incidence by sex and sport classification. J Athl Train.

[24] Knottnerus A, Tugwell P (2008). STROBE--a checklist to strengthen the reporting of observational studies in epidemiology. J Clin Epidemiol.

[25] Hagiwara G, Isogai H (2013). The study of the athletic identity: reexamination of the Japanese athletic identity measurement scale, and comparison of the competitive level and student athlete. J Phys Exerc Sports Sci.

[26] Hagiwara G, Isogai H (2014). Examining the commitment for competitive sports: development of Japanese version of sports commitment scale. Japanese J Sport Psychol.

[27] Woby SR, Roach NK, Urmston M, Watson PJ (2005). Psychometric properties of the TSK-11: a shortened version of the Tampa scale for Kinesiophobia. Pain.

[28] Kikuchi N, Matsudaira K, Sawada T, Oka H (2015). Psychometric properties of the Japanese version of the Tampa scale for Kinesiophobia (TSK-J) in patients with whiplash neck injury pain and/or low back pain. J Orthop Sci.

[29] Webster KE, Feller JA, Lambros C (2008). Development and preliminary validation of a scale to measure the psychological impact of returning to sport following anterior cruciate ligament reconstruction surgery. Phys Ther Sport.

[30] Hirohata K, Aizawa J, Furuya H, Mitomo S, Ohmi T, Ohji S, Ohara T, Koga H, Yagishita K, Webster KE (2020). The Japanese version of the anterior cruciate ligament-return to sport after injury (ACL-RSI) scale has acceptable validity and reliability. Knee Surg Sports Traumatol Arthrosc.

[31] Ardern CL, Taylor NF, Feller JA, Whitehead TS, Webster KE (2015). Sports participation 2 years after anterior cruciate ligament reconstruction in athletes who had not returned to sport at 1 year: a prospective follow-up of physical function and psychological factors in 122 athletes. Am J Sports Med.

[32] Ardern CL, Webster KE, Taylor NF, Feller JA (2011). Return to the preinjury level of competitive sport after anterior cruciate ligament reconstruction surgery: two-thirds of patients have not returned by 12 months after surgery. Am J Sports Med.

[33] Kvist J, Ek A, Sporrstedt K, Good L (2005). Fear of re-injury: a hindrance for returning to sports after anterior cruciate ligament reconstruction. Knee Surg Sports Traumatol Arthrosc.

[34] Han F, Banerjee A, Shen L, Krishna L (2015). Increased compliance with supervised rehabilitation improves functional outcome and return to sport after anterior cruciate ligament reconstruction in recreational athletes. Orthop J Sports Med.

[35] Brewer BW, Cornelius AE: Self-protective changes in athletic identity following anterior cruciate ligament reconstruction. Psychol Sport Exerc 2010, 11(1):1–5, 1, DOI: 10.1016/j.psychsport.2009.09.005. 10.1016/j.psychsport.2009.09.005 PMC2783627 20161402

[36] Lamont-Mills A, Christensen SA (2006). Athletic identity and its relationship to sport participation levels. J Sci Med Sport.

[37] Grindem H, Snyder-Mackler L, Moksnes H, Engebretsen L, Risberg MA (2016). Simple decision rules can reduce reinjury risk by 84% after ACL reconstruction: the Delaware-Oslo ACL cohort study. Br J Sports Med.

[38] Christakou A, Lavallee D (2009). Rehabilitation from sports injuries: from theory to practice. Perspect Public Health.

[39] Wayda VK, Armenth-Brothers F, Boyce BA (1998). Goal setting: a key to injury rehabilitation. Int J Athl Ther Train.

[40] Greenberg EM, Greenberg ET, Albaugh J, Storey E, Ganley TJ (2019). Anterior cruciate ligament reconstruction rehabilitation clinical practice patterns: a survey of the PRiSM society. Orthop J Sports Med.

[41] Greenberg EM, Greenberg ET, Albaugh J, Storey E, Ganley TJ (2018). Rehabilitation practice patterns following anterior cruciate ligament reconstruction: a survey of physical therapists. J Orthop Sports Phys Ther.

